# Distribution characteristics and diversity of myxomycetes in three parallel rivers in Yunnan, China

**DOI:** 10.1371/journal.pone.0293260

**Published:** 2024-01-02

**Authors:** Xiangyang Zhu, Odeshnee Naicker, Zhanwu Peng, Bao Qi, Qi Wang, Yu Li

**Affiliations:** 1 Engineering Research Centre of Chinese Ministry of Education for Edible and Medicinal Fungi, College of Plant Pathology, Jilin Agricultural University, Changchun, Jilin Province, China; 2 Forestry College, Beihua University, Jilin, Jilin Province, China; 3 Department of Plant and Soil Sciences, University of Venda, Thohoyandou, South Africa; 4 Information Center, Jilin Agricultural University, Changchun, Jilin Province, China; PLOS: Public Library of Science, UNITED STATES

## Abstract

Three Parallel Rivers is one of the world’s biodiversity hotspots. However, the research on myxomycetes diversity is scarce in this area. Random sampling was used to investigate myxomycetes’ diversity and distribution characteristics in this area. One hundred and seventeen species, including three varieties, were obtained, belonging to 28 genera, nine families, and six orders, with *Arcyria cinerea* and *Physarum viride* being the dominant species. Moreover, four species and one variety were first reported in China. Twenty-six species and one variety were first reported in Yunnan Province. The species’ most commonly utilized substrate for fruiting bodies was decaying wood, and *Cribraria* was the dominant genus. The species diversity was most abundant in mixed broadleaf-conifer forests. Species similarity between coniferous and broad-leaved forests was much higher than the pairwise comparison of other forest types. NMDS analysis shows that substrate and forest types had insignificant effects on myxomycetes communities, while river valley had a significant effect. The myxomycetes community similarity between river valleys is unrelated to geographical proximity.

## Introduction

Myxomycetes are classified as eukaryote protozoans and are often studied by mycologists. Their life cycles have a dynamic plasmodial stage, and they feed on bacteria while also serving as a food source for insects. They can exist in many ecological environments, such as arid deserts [1–3] (which are always studied by moist chamber culture, since fruiting bodies are rarely found in those environments), snowmelt zones with snow [4–7], and most commonly, forests [8–11]. There are also accounts of some aquatic habitats containing different species of myxomycetes [12, 13]. Accordingly, myxomycetes are considerably diverse and occur in various environmental conditions.

Some myxomycetes can fruit on decaying wood, bark, ground litter, dung, and even on the surface of stones or garbage. Not all species can fruit on all kinds of substrates, and it seems that some unique species are present in each ecological environment [14]. One group of coprophilous myxomycetes, with a wide distribution, fruit on the dung of herbivorous animals containing abundant nutrients [15–19]. Researchers have previously worked on the different substrate types of myxomycetes via moist chamber culture [20, 21]. Rojas and Stephenson (2007) [22] reported on the distribution of myxomycetes in high-altitude oak forests in Costa Rica. Most fruiting bodies collected from oak forests above 3000 m in the Talamanca Mountains were found on decaying wood, twigs, and bryophytes, while a small portion was found on litter. Some species also show selectivity to substrates in deserts [2, 23], although they are all arid deserts, a species that primarily uses cacti as a substrate is very rare and was observed in the Namib Desert (Namibia) [2]. Moreover, many discoveries made in the North and South American deserts and some species found outside the American continents were related to the distribution of this host plant [24]. The structure of the fruiting bodies also showed an association with the environment. Lado (2004) [25] observed that most of the fruiting bodies of Nivicolous myxomycetes have a white calcareous shell and a membranous peridium. These morphological modifications can be explained as being a form of protective shielding possibly used during sporulation.

Based on the living substrates of myxomycete are most of the plant residues, the researches of species distributed in a forest ecosystem are deductively common. Eliasson [26] investigated the pattern of occurrence of myxomycetes in a spruce forest in South Sweden, and several species showed apparent substrate preferences in this coniferous forest. Stephenson [27] investigated the assemblage of myxomycetes associated with decaying fronds of nikau palm in New Zealand, where most species belong to Physarales and Trichiales. In terms of species richness and diversity, Li [28] and Gao [11] found that mixed broadleaf-conifer forest was much higher than broadleaf and coniferous forests.

The "Three Parallel Rivers" is located in the western part of Yunnan Province. It is the rift zone of the Hengduan Mountains from the southern extension of the Qinghai-Tibet Plateau to the Yunnan-Guizhou Plateau. Nujiang River, Lancang River, and Jinsha River are parallel, forming a unique geography wonder in the world. Additionally, the terrain is high in the North and low in the South; the highest peak is Kawakarpo, Mt. Meili is more than 6000 m high [29] and is located in northwestern Yunnan within the geographic scope of our research. Subtropical and alpine temperate climates coexist in this area, along with extensive ranges in elevation and the alternation between southwesterly and southeasterly monsoons resulting in wet (May to Oct) and dry (Nov to Apr) periods [30]. The vegetation can be generally divided into 5 broad types in this area: Savanna (1900–3000 m), Mixed broadleaf-conifer forests (3000–3800 m), the Abies forests (3800–4100 m), Alpine shrubs (4000–4350 m), and Alpine meadows (4350–4500 m) [31]. There were reports on the investigation of myxomycetes conducted in Yunnan at the beginning of the 21st century [32, 33], and also some accounts of myxomycetes in the tropical areas of Yunnan in recent years [34, 35], however, there were few studies focused in Three Parallel Rivers. Therefore, in this study, our purpose was to investigate myxomycetes resources in Three Parallel Rivers, one of the main parts in Yunnan, add new records of myxomycetes for the area, and establish a relationship between species and substrates.

## Materials and methods

### Specimen collection and organization

All specimens observed in this study were collected in “Three Parallel Rivers” of Yunnan Province and from a single location in Tibet, from Aug to Sep 2019. Collection site coordinates were from 98°28′57.41″ to 102°49′44.91″ E and 24°41′18.67″ to 29°16′18.35″ N, and forest types included coniferous, broad-leaved, and mixed broadleaf-conifer, altitudes ranged from 1103 m to 4135 m (Table 1). The average temperature of this area was around 20°C, with less rainfall, but some high altitudes had high humidity. Using a randomized sampling method, we usually collected in the forest parks or natural reserves of the “Three Parallel Rivers” area, and the substrate layer of forests is enough thicker which suitable for the growth of myxomycetes; we walked through the trail of the forest and collected whatever myxomycetes were present. One collection site was investigated one time except Biluo Snow Mountain (Two times), Mangkang (Three times), Baima Snow Mountain (Two times) in these two months, and all collection sites were open for collectors. All collected samples were covered with aluminum foil and placed in boxes with fixed partitions. To obtain sufficiently complete fruiting bodies, avoiding violent vibration and excessive squeezing during storing was imperative. In addition, we added color-changing silica gel beads to the box to dry the wet specimens, which were replaced as necessary. All specimens were then sorted, fixed in boxes on paper, and stored at the Engineering Research Center of the Ministry of Education of Jilin Agricultural University. Each specimen was labeled accordingly and had detailed collection information.

**Table 1:** Records of collection sites at different altitude intervals.

Location	Longitude	Latitude	Elevation (m)	Forest type
Gaoligong Mountain Nature Reserve (GMNR)	98°46′3.13″	24°49′48.57″	2165	BF
Laifeng Mountain (LFM)	98°28′57.41″	25°1′13.35″	1737	CBF
Gaoligong Mountain Nature Park (GMNP)	98°43′5.74″	25°26′54.89″	1986	BF
Fugongdangxiao (FGDX)	98°51′42.43″	26°54′23.47″	1184	CBF
Emadi (EMD)	98°51′42.07″	26°54′10.28″	1103	CBF
Baizexu (BZW)	98°51′1.64″	26°53′24.31″	1887	CBF
Taibao Mountain (TBM)	99°9′6.46″	25°7′22.49″	1790	CBF
Biluo Snow Mountain (BLSM)	99°4′56.65″	26°55′17.34″	2403	CF
	99°8′22.99″	26°46′59.51″	2396	CF
Shualao (SL)	98°46’5.73"	28°5’4.87″	3462	CF
	98°47′51.61″	28°8′43.58″	3010	CF
Cangshan Mountain (CSM)	100°8’55.81″	25°40’5.89″	2299	CBF
Jizu Mountain (JZM)	100°23′52.62″	25°56′59.82″	1897	CBF
Xinshengqiao National Forest Park (XSQ)	99°21′1.7″	26°35′55.43″	2639	CF
Yunnan Golden Monkey National Forest Park (YGM)	99°22′29.34″	27°36′41.44″	2179	CF
Laojun Mountain (LJM)	99°46′33.11″	26°39′9.03″	3454	CF
Jiguan Mountain (JGM)	99°16′12.6″	27°11′9.19″	2224	CF
Baima Snow Mountain (BMSM)	99°7′33.11″	28°19′9.65″	3872	BF
	99°10′12.14″	28°17′57.27″	3317	BF
Mangkang (MK) (Tibet)	98°39′22.41″	29°11′45.05″	3582	CF
	98°40′29.94″	29°13′31.39″	3849	CF
	98°40′29.09″	29°16′18.35″	4135	CF
Yulong Snow Mountain (YLSM)	100°15′0.86″	27°6′36.87″	3041	CF
Tiger Leaping Gorge (TLG)	100°3′58.21″	27°10′19.44″	1872	BF
Qianhu Mountain Nature Reserve (QHM)	99°49′39.81″	27°24′9.38″	3526	CF
Pudacuo National Forest Park (PDC)	99°54′34.26″	27°48′8.99″	3383	CF
Denggao Mountain (DGM)	101°38′58.93″	24°41′18.67″	2031	CBF
Zixi Mountain (ZXS)	101°24′9.47″	25°1′9.08″	2400	CBF
Yuntai Mountain (YTM)	101°17′32.7″	25°17′37.69″	2046	CF
Jichong Mountain (JCM)	102°49′44.91″	25°1′4.9″	1951	CBF
Qipan Mountain (QPM)	102°34′48.16″	25°3′4.1″	2439	CBF

Notes: CF: coniferous forest, BF: broad-leaved forest, CBF: mixed broadleaf-conifer forest.

### Identification

Test materials were fixed on glass slides with 94.00% alcohol, followed by adding 3.00% KOH dropwise until the contraction effect due to alcohol was corrected. Before placement of the cover glass, one drop of 8.00% glycerin was added. Fruiting bodies and microscopic structures were observed using light microscopy. We observed 20 sporocarps under the Leica M125 (Weztlar, Germany) stereomicroscope and measured 20 spores and 10 lime nodes under the Leica DM2000 (Weztlar, Germany) optical microscope. We then compared the morphological characteristics of our organisms with Martin and Alexopopulos’s descriptions [36]. The nomenclature followed Lado (2005–2023) [37]. The classification was also based on Leontyev’s research [38].

### Data analysis of species biodiversity

Relative abundance (RA) determinations were characterized as the percentage composition of a particular myxomycete in the sample relative to the total number of samples [39]. The diversity index (*H*′) was calculated using the Shannon-wiener formula: *H*′ = −Σ*Pi* ln*Pi*, where *Pi* represented the relative abundance (RA) [39]. The Evenness was calculated using the formula: *E* = *H*′/*H*′_*max*_, *H*′_*max*_ = ln*S*, *S* was the number of total species [40]. The coefficient of community index (CC) was calculated using the Sorensen formula [41]: CC = 2z/(x+y), where x was the number of species in X, y was the number of species in Y, and z was the number of species in common between the two places. The S/G ratio was also used in this study to indicate taxonomic diversity, where S was the number of species in all genera and G was the number of genera. If the S/G value was low, the inversely proportional taxonomic diversity was higher [42]. The species rank curve was conducted by “ggplot2” R-package 3.4.0 software [43]. Nonmetric multidimensional scaling (NMDS) based on Bray-Curtis distances was performed on the Tutools platform (https://www.cloudtutu.com), a free online data analysis website. The R-packages “Vegan” was used to perform a Permutational multivariate analysis of variance (Permanova) to detect the influence of different substrate and forest types on the myxomycete community [44].

### Distribution of myxomycetes on different substrates

We determined which substrates all the collected myxomycetes utilized; these were then divided into different categories, namely: decayed woods (Dw) (Usually collected from the decay trunk of fallen trees and tree stumps, their degree of decay and water holding capacity is higher than that of branches), fallen leaves (Fl), barks (Ba), branches (Br) (Much smaller and more rigid than Dw), Others: included mosses, grasses, pine needles, live grasses, litters (indistinguishable plant tissues) and stones. Next, the number of myxomycetes on each substrate was counted, then shared and unique species were compared by jvenn online (http://jvenn.toulouse.inra.fr/app/example.html). Finally, the number and percentage of species on each substrate were calculated; the dominant genus included the most abundant species and percentage.

### Distribution of myxomycetes in different forest types

Collection areas were sorted according to different forest types: coniferous forest, broad-leaved forest, and mixed broadleaf-conifer forest, the collection sites belonging to each forest type are reflected in Table 1. We counted the number of species at each forest type, and the common species; the unique species at different forest types were then compared by jvenn online. The statistical method used was the same for “Classification of myxomycete substrates” at the genus levels.

### Division of river valleys in Three Parallel Rivers

The collection area was divided into four sections: Nujiang River, Lancang River, and Jinsha River, named West of Nujiang River, Lancang River Valley, Jinsha River Valley and East of Jinsha River. The collection sites in each section are shown in Table 2.

**Table 2:** Collection sites in different river valleys.

Location	Collection sites
West of Nujiang River (WNR)	GMNR, LFM, GMNP, EMD, BZW, FGDX
Lancang River Valley (LRV)	TBM, BLSM, SL
Jinsha River Valley (JRV)	CSM, JZM, XSQ, YGM, LJM, JGM, BMSM, MK
East of Jinsha River (EJR)	YLSM、TLG、QHM、PDC

## Results

### Composition of myxomycetes collected from the Three Parallel Rivers

**Fig 1:**
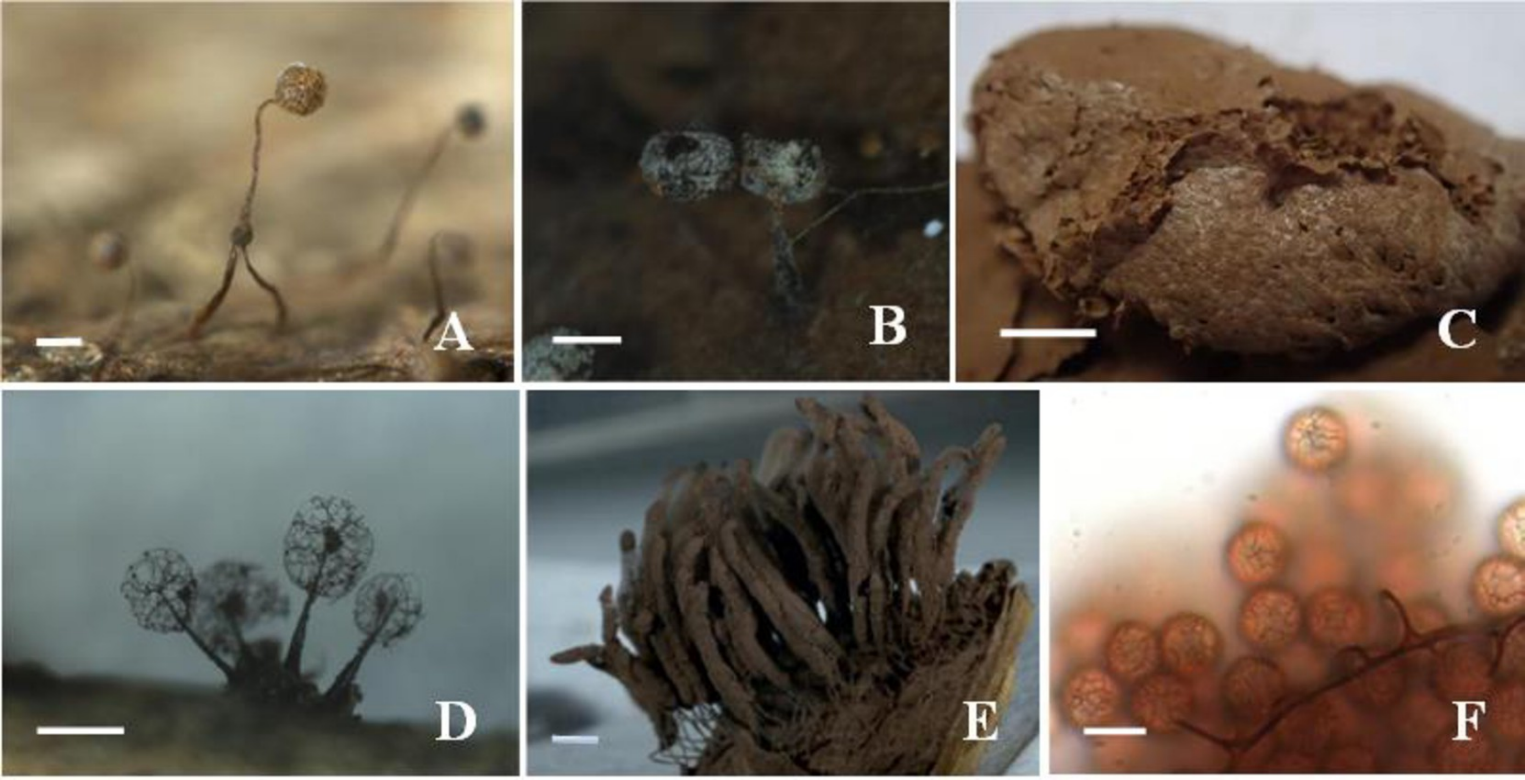
Four new record species and one new record variety in China. A: Sporocarps of *Cribraria stellifera*, B: Sporocarps of *Didymium columellacavum*, C: Pseudoaethalia of *Tubifera applanata*, D: Sporocarps of *Macbrideola synsporos*, E: Sporocarps of *Stemonitopsis hyperopta* var. *landewaldii*, F: Spores of *Stemonitopsis hyperopta* var. *landewaldii*: the spores are dark and the size of this variety are 7–9 μm which bigger than *Stemonitopsis hyperopta*, spore ornamentation is weak and irregular. Bars: A: 1 mm, B: 0.5 mm, C: 1 mm, D: 0.5 mm, E: 1 mm, F: 8 μm.

**Fig 2:**
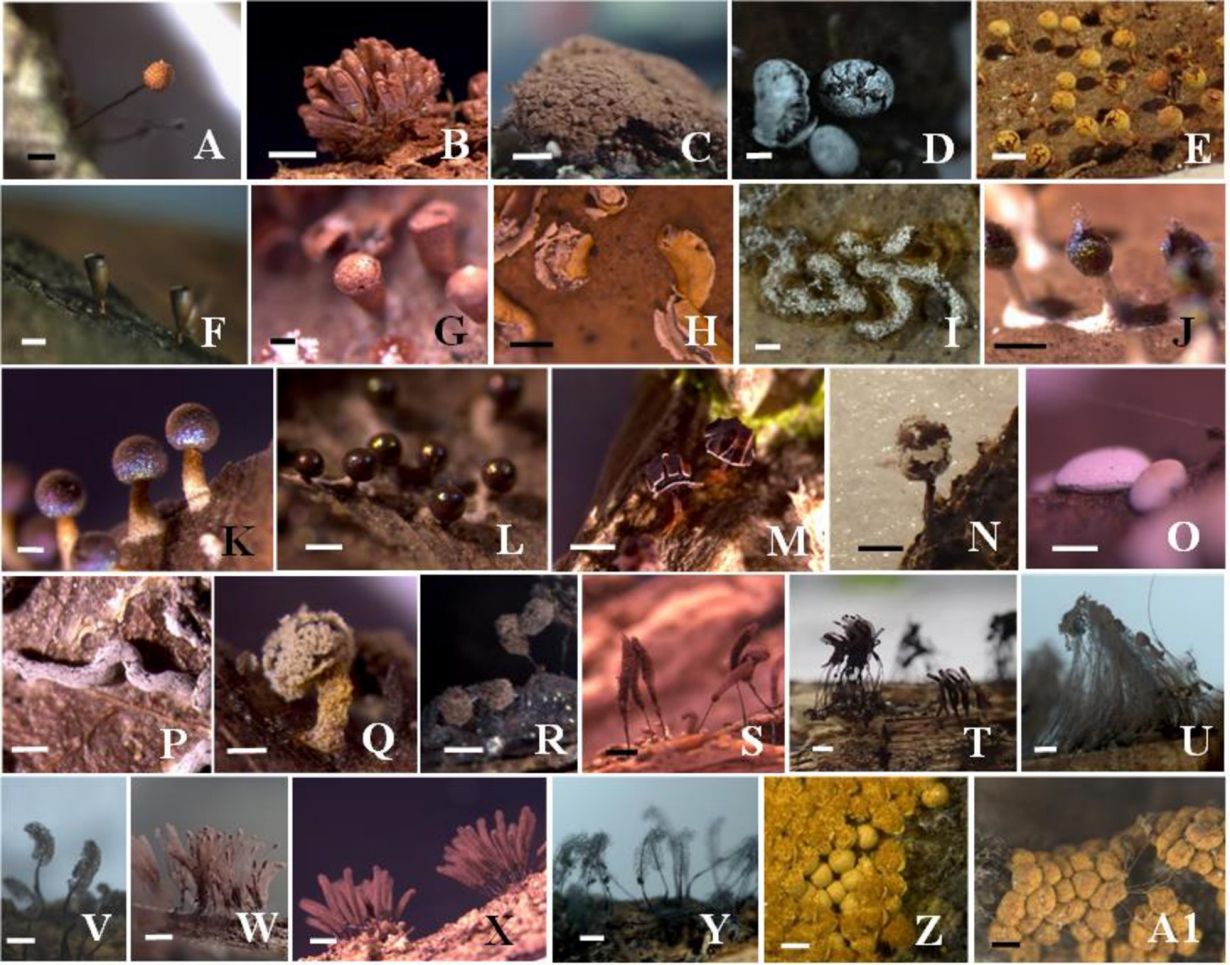
Twenty-six new records species and one new record variety in Yunnan Province. A: *Cribraria tenella*, B: *Tubifera dimorphotheca*, C: *Tubifera montana*, D: *Badhamia macrospora*, E: *Craterium aureum*, F: *Craterium minutum* var. *brunneum*, G: *Cra*. *reticulatum*, H: *P*. *bogoriense*, I: *P*. *plicatum*, J: *Diachea bulbillosa*, K: *Dia*. *splendens*, L: *Dia*. *subsessilis*, M: *Did*. *radiatum*, N: *Did*. *rugosum*, O: *D*. *comatum*, P: *D*. *flexuosum*, Q: *Didymium leoninum*, R: *Comatricha pellucida*, S: *Comatricha fragilis*, T: *Stem*. *irregularis*, U: *Stemonaria laxiretis*, V: *Stem*. *fuscoides*, W: *Stem*. *reticulospora*, X: *S*. *marjana*, Y: *Sym*. *flaccidus*, Z: *Trichia affinis*, A1: *T*. *persimilis*. Bars: A, D, G, I, J, L, N, O-V, A1: 0.5 mm, B, E, M, W-Z: 1 mm, C: 2 mm, F: 0.2 mm, H: 1.5 mm, K: 0.1 mm.

Morphological identification of 776 specimens of myxomycetes belonged to six orders, nine families, 28 genera, 117 species, including three varieties. Four species and one variety were first recorded in China (Marked with “**”, Fig 1, Table 3), 26 species and one variety were first recorded in Yunnan (Marked with “*”, Fig 2, Table 3). *Arcyria cinerea* and *Physarum viride* are the dominant species in this area, having relative abundance (RA) values of 7.44% and 6.67%, respectively, with 58 and 52 samples collected. Of the samples collected, 25–49 samples (RA range from 3% to 6%) were collected for 4 species, 8–24 samples (RA range from 1% to 3%) were collected for 26 species, 3–7 samples (RA range from 0.3% to 1%) were collected for 31 species, 1–2 samples (RA range from 0.1% to 0.3%) were collected for 54 species which were rare in this area (Table 4). The diversity index (*H*′) = 4.13, indicates that myxomycetes are highly abundant in this area.

**Table 3:** All myxomycetes species collected in Three Parallel Rivers.

Genus	Species	Substrates	Altitudes	Forest types	Records
*Cribraria*	*C*. *argillacea*	Dw	B, C	a, b	16
	*C*. *atrofusca*	Dw	C	a	1
	*C*. *aurantiaca*	Dw, Ba	A, B, C	a, b, c	10
	*C*. *cancellata*	Dw, Litters	A, B, C	a, b, c	29
	*C*. *media*	Dw	C	c	13
	*C*. *microcarpa*	Dw	B	a	1
	*C*. *mirabilis*	Dw, Mosses	A, B, C	a, b, c	15
	*C*. *persoonii*	Dw	B	a	4
	*C*. *piriformis*	Dw, Mosses	B, C	a, b	9
	*C*. *purpurea*	Dw	C	a	8
	*C*. *splendens*	Dw, Ba, Mosses	A, B, C	a, b, c	11
	**C. *stellifera*	Dw	C	a	3
	*C. *tenella*	Dw	A	c	1
	*C*. *vulgaris*	Dw, Mosses	B, C	a, b	9
*Licea*	*Lic*. *minima*	Dw	B	c	1
*Lycogala*	*Lyc*. *epidendrum*	Dw	C	a, b	13
	*Lyc*. *conicum*	Dw	C	a	1
*Tubifera*	**Tub. *applanata*	Ba, Fl	B	c	2
	*Tub. *dimorphotheca*	Dw	A	b	3
	*Tub*. *ferruginosa*	Dw, Mosses	B, C	a, c	6
	*Tub. *montana*	Dw, Mosses	C	b	3
*Badhamia*	*B. *macrospora*	Dw	C	a	1
*Craterium*	*Cra. *aureum*	Dw, Fl, Mosses	C	a	4
	*Cra*. *leucocephalum*	Br, Fl, Pine needles, Litters, Grasses	A, B	a, c	21
	*Cra*. *leucocephalum* var. *cylindricum*	Fl, Litters	A	c	4
	*Cra*. *minutum*	Dw, Br, Fl, Stones	A, B, C	a, c	8
	*Cra. *minutum* var. *brunneum*	Fl	B	c	1
	*Cra. *reticulatum*	Ba, Fl	B, C	c	7
*Fuligo*	*F*. *septica*	Dw	A	c	1
*Leocarpus*	*Leo*. *fragilis*	Dw, Ba, Br, Fl, Grasses	C	a	8
*Physarum*	*P*. *album*	Dw, Ba, Br, Mosses	A, B, C	a, b, c	38
	*P*. *auriscalpium*	Fl	B, C	c	2
	*P*. *bivalve*	Dw, Fl, Br	A, B, C	a, c	9
	*P. *bogoriense*	Fl	B	c	1
	*P*. *citrinum*	Ba, Fl	A, B	c	4
	*P*. *didermoides*	Litter	B	c	1
	*P*. *galbeum*	Ba	C	a	1
	*P*. *leucophaeum*	Dw	A	c	2
	*P*. *leucopus*	Fl	B	a	1
	*P*. *melleum*	Dw, Fl, Litters	A, B, C	a, b, c	14
	*P*. *newtonii*	Fl	B	a	1
	*P*. *oblatum*	Fl	B	c	2
	*P*. *ovisporum*	Fl, Litters	A, C	a, c	2
	*P. *plicatum*	Fl	B	c	1
	*P*. *robustum*	Br	B	b	1
	*P*. *roseum*	Dw	A	c	1
	*P*. *viride*	Dw, Ba, Br, Mosses	A, B, C	a, b, c	52
*Willkommlangea*	*W*. *reticulata*	Dw	C	b	3
*Diachea*	*Dia. *bulbillosa*	Fl	A, B	a, c	2
	*Dia*. *leucopodia*	Dw, Ba, Br, Fl, Live grasses	A, B	a, c	11
	*Dia. *splendens*	Fl	A, B	b, c	2
	*Dia. *subsessilis*	Fl	B	a	1
*Diderma*	*Did*.* effusum*	Fl	B, C	a, c	2
	*Did*. *hemisphaericum*	Br, Fl	A, B	b, c	4
	*Did. *radiatum*	Dw	C	c	2
	*Did. *rugosum*	Br	B	c	1
*Didymium*	**D. *columellacavum*	Fl, Pine needles	A, C	a, c	2
	*D. *comatum*	Grasses	A	c	2
	*D*. *crustaceum*	Dw	B	c	5
	*D*. *difforme*	Fl, Pine needles	B	c	2
	*D. *flexuosum*	Fl	A	c	2
	*D. *leoninum*	Fl	B	c	6
	*D*. *megalosporum*	Ba, Fl, Br, Pine needles, Stones	A, B, C	a, c	26
	*D*. *minus*	Fl, Pine needles	C	a	3
	*D*. *nigripes*	Dw, Ba, Br, Fl, Pine needles, Stones	A, B, C	a, b, c	25
	*D*. *squamulosum*	Dw, Br, Fl, Pine needles	A, B, C	a, c	10
	*D*. *verrucosporum*	Fl	C	a	1
*Lepidoderma*	*Lep*. *tigrinum*	Dw, Mosses	C	c	1
*Lamproderma*	*Lam*. *scintillans*	Dw, Ba, Fl	A, B, C	a, b, c	17
*Comatricha*	*Com. *fragilis*	Dw	C	c	2
	*Com*. *laxa*	Dw	B, C	a, b, c	10
	*Com*. *nigra*	Dw	C	a, b	3
	*Com. *pellucida*	Litter	B	a	1
	*Com*. *pulchella*	Br, Fl	A, C	c	2
	*Com*. *tenerrima*	Dw, Ba	B	b, c	2
*Enerthenema*	*E*. *papillatum*	Dw	A, B	c	5
*Collaria*	*Col*. *arcyrionema*	Dw	C	a	1
*Macbrideola*	**M. *synsporos*	Dw	C	a	1
*Stemonaria*	*Stem. *fuscoides*	Dw	B	a	2
	*Stem. *irregularis*	Dw	B	c	5
	*Stem. *laxiretis*	Dw	C	b	1
	*Stem*. *longa*	Dw, Ba	B	b	1
	*Stem. *reticulospora*	Dw	C	b	1
*Stemonitis*	*S*. *axifera*	Dw	A, C	a, b, c	5
	*S*. *flavogenita*	Dw	A, C	a, b	3
	*S*. *fusca*	Dw, Ba	B	c	4
	*S. *marjana*	Ba	A	c	2
	*S*. *smithii*	Moss	C	a	1
	*S*. *splendens*	Dw, Ba	A, B, C	b, c	11
*Stemonitopsis*	**Ste. *hyperopta* var. *landewaldii*	Dw	C	b	3
	*Ste*. *typhina*	Dw	A, B, C	a, b, c	10
*Symphytocarpus*	*Sym. *flaccidus*	Dw	C	c	1
*Arcyodes*	*Arc*. *incarnata*	Dw, Ba	C	a	4
*Arcyria*	*A*. *cinerea*	Dw, Ba, Br	A, B, C	a, b, c	58
	*A*. *denudata*	Dw	A, B, C	a, b, c	12
	*A*. *globosa*	Fl	A	b	1
	*A*. *incarnata*	Dw, Ba	C	a, b	6
	*A*. *insignis*	Dw	A	c	1
	*A*. *obvelata*	Dw	B, C	a, c	2
	*A*. *occidentalis*	Ba	C	c	3
	*A*. *oerstedii*	Dw, Fl, Mosses	A, B	b, c	8
	*A*. *pomiformis*	Dw	B, C	a, b, c	5
	*A*. *versicolor*	Dw	B, C	a, c	2
*Hemitrichia*	*H*. *calyculata*	Dw, Ba, Mosses	A, B, C	a, b, c	23
	*H*. *clavata*	Dw, Mosses	A, C	a, b, c	15
	*H*. *serpula*	Dw, Ba, Br, Fl, Mosses	A, B, C	a, b, c	22
*Perichaena*	*Per*. *depressa*	Ba	A, B, C	a, c	9
	*Per*. *vermicularis*	Fl	A	c	6
*Trichia*	*T. *affinis*	Dw	C	c	3
	*T*. *botrytis*	Dw, Fl	C	a, b	5
	*T*. *decipiens*	Dw	C	a, b, c	22
	*T*. *favoginea*	Dw, Ba	A, C	a, c	7
	*T*. *lutescens*	Ba	C	a	2
	*T. *persimilis*	Dw, Ba	A, C	a, c	3
	*T*. *scabra*	Ba	B	c	1
	*T*. *subfusca*	Ba	C	a	1
	*T*. *varia*	Ba	C	a	1

Notes: Dw: decayed woods, Fl: fallen leaves, Ba: barks, Br: branches; A: 1000–2000 m, B: 2000–3000 m, C: 3000–4200 m; a: CF, b: BF, c: CBF

**Table 4:** Relative abundance of myxomycetes species in Three Parallel Rivers.

Species copies (relative abundance)	Species	Number
>50 (6%-8%)	*A*. *cinerea*, *P*. *viride*	2
25–49 (3%-6%)	*P*. *album*, *C*. *cancellatum*, *D*. *megalosporum*, *D*. *nigripes*	4
8–24 (RA = 1%-3%)	*H*. *calyculata*, *H*. *serpula*, *T*. *decipiens*, *Cra*. *leucocephalum*, *Lam*. *scintillans*, *C*. *argillacea*, *C*. *mirabile*, *S*. *splendens*, *H*. *clavata*, *P*. *melleum*, *L*. *epidendrum*, *C*. *media*, *A*. *denudata*, *C*. *splendens*, *Dia*. *leucopodia*, *Ste*. *typhina*, *D*. *squamulosum*, *C*. *aurantiaca*, *Com*. *laxa*, *C*. *vulgaris*, *C*. *pirformis*, *Per*. *depressa*, *C*. *purpurea*, *Cra*. *minutum*, *Leo*. *fragilis*, *A*. *oerstedii*	26
3–7 (RA = 0.3%-1%)	*Cra*. *reticulatum*, *T*. *favoginea*, *Tub*. *ferruginosa*, *P*. *bivalve*, *D*. *leoninum*, *Per*. *vermicularis*, *A*. *incarnata*, *S*. *axifera*, *Stem*. *irregularis*, *E*. *papillatum*, *D*. *crustaceum*, *A*. *pomiformis*, *T*. *botrytis*, *C*. *persoonii*, *S*. *fusca*, *Com*. *nigra*, *P*. *citrinum*, *Cra*. *aureum*, *Cra*. *leucocephalum* var. *cylindricum*, *Did*. *hemisphaericum*, *Arc*. *incarnata*, *C*. *stellifera*, *Ste*. *hyperopta* var. *landewaldii*, *Tub*. *montana*, *Tub*. *dimorphotheca*, *W*. *reticulata*, *D*. *minus*, *T*. *affinis*, *T*. *persimilis*, *A*. *occidentalis*, *S*. *flavogenita*	31
1–2 (RA = 0.1%-0.3%)	*A*. *obvelata*, *S*. *marjana*, *Com*. *pulchella*, *Com*. *fragilis*, *Com*. *tenerrima*, *Stem*. *fuscoides*, *Tub*. *applanata*, *P*. *auriscalpium*, *P*. *ovisporum*, *P*. *leucophaeum*, *P*. *citrinum*, *P*. *roseum*, *D*. *flexuosum*, *D*. *difforme*, *D*. *columellacavum*, *D*. *comatum*, *Did*. *radiatum*, *Did*.* effusum*, *Dia*. *bulbillosa*, *Dia*. *splendens*, *A*. *versicolor*, *T*. *lutescens*, *C*. *tenella*, *C*. *atrofusca*, *S*. *smithii*, *Stem*. *longa*, *Stem*. *laxiretis*, *Stem*. *reticulospora*, *Sym*. *flaccidus*, *P*. *robustum*, *P*. *didermoides*, *P*. *plicatum*, *P*. *galbeum*, *P*. *leucopus*, *P*. *bogoriense*, *P*. *newtonii*, *Cra*. *minutum* var. *brunneum*, *Col*. *arcyrionema*, *D*. *verrucosporum*, *D*. *rugosum*, *Lep*. *tigrinum*, *A*. *insignis*, *A*. *globosa*, *T*. *scabra*, *T*. *subfusca*, *T*. *varia*, *Lic*. *minima*, *B*. *macrospora*, *M*. *synsporos*, *F*. *septica*, *Com*. *pellucida*, *Dia*. *subsessilis*, *Lyc*. *conicum*, *C*. *microcarpa*	54

### Myxomycetes substrate types at the genus level

**Fig 3:**
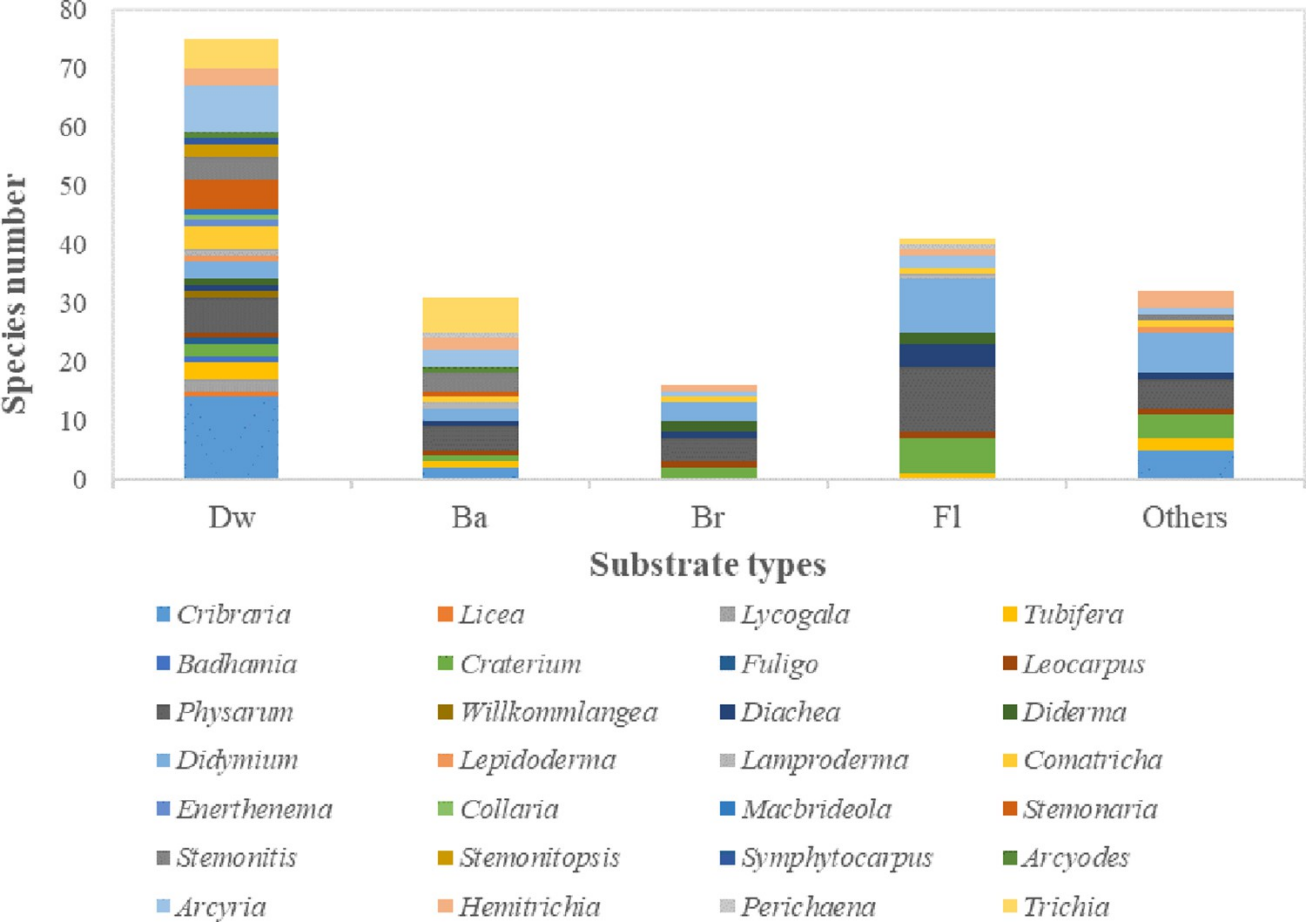
The genus level distribution at different substrate types.

At the genus level (Fig 3, Table 5), 27 genera of myxomycetes produced fruiting bodies on Dw; only *Perichaena* was not observed. Ten genera of myxomycetes were found on Br, 16 on Ba, 11 on Fl, and eleven genera were noted on Others (Mosses, Grasses, Pine needles, Live Grasses, Litters, and Stones). Consequently, Dw contained the most abundant genera of all kinds of substrate (S1 Table).

**Table 5:** Dominant genera of myxomycetes on different substrates.

Substrates	Dominant genus	Species number	Genus number	Total species	Percent (%)	*H’*	*E*
**Dw**	*Cribraria*	14	27	75	18.67	3.73	0.86
**Ba**	*Trichia*	6	16	31	19.35	2.57	0.93
**Br**	*Physarum*	4	9	16	25.00	3.01	0.88
**Fl**	*Physarum*	11	13	41	26.83	3.21	0.86
**Others**	*Didymium*	7	11	32	21.88	3.28	0.95

**Fig 4:**
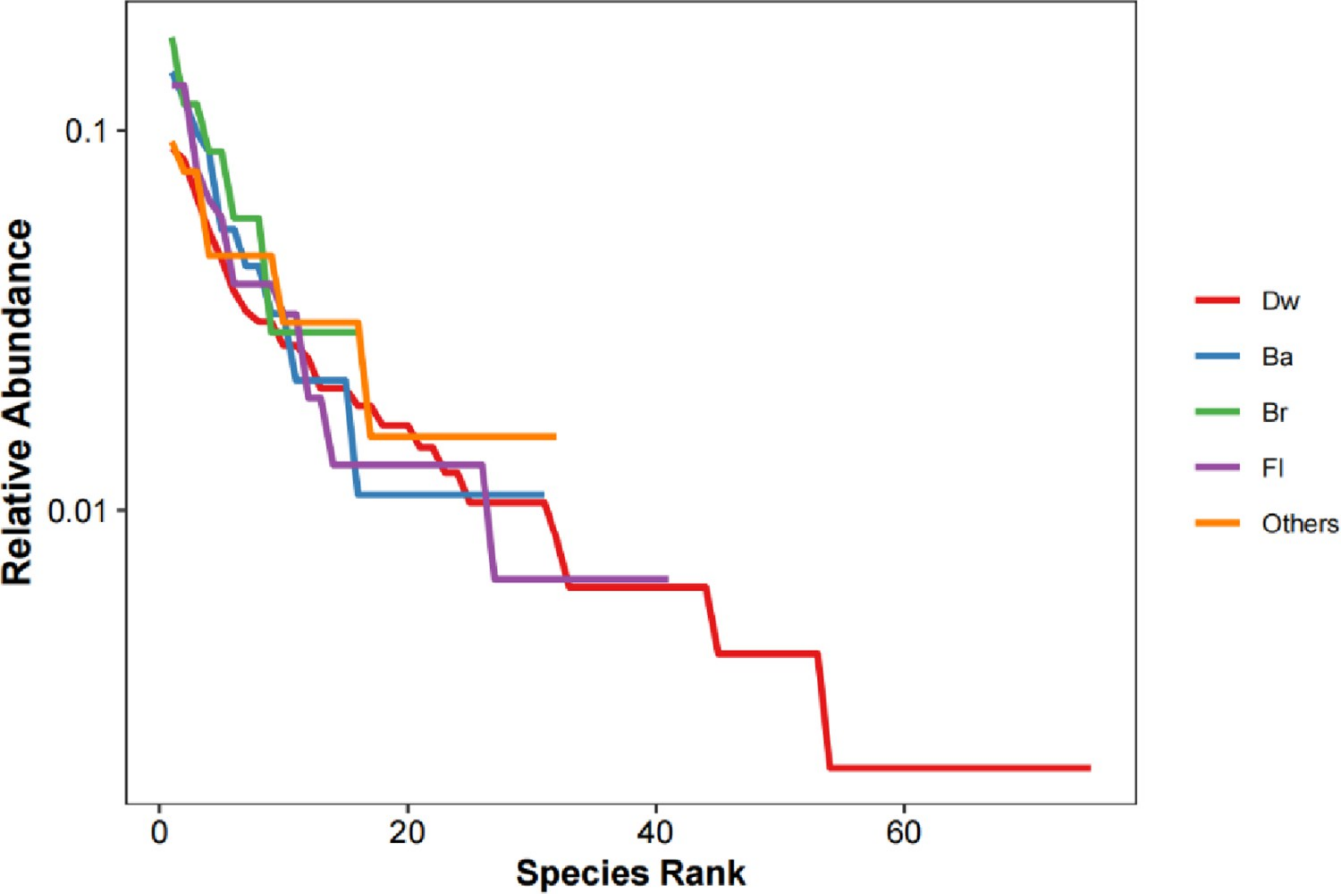
The species rank curve on different substrates.

Fourteen species of the genus *Cribraria* produced fruiting bodies on Dw, accounting for 18.67% of all species present, indicating that it was the dominant genus for this type of substrate. Four species of myxomycetes in *Physarum* fruited on Br, accounting for 25.00% of all myxomycetes inhabiting the substrate, while in terms of the number of species, the genus *Didymium* was the only species observed less than *Physarum*. The genus *Trichia* was dominant on Ba, including six species, accounting for 19.35%, while the dominant genus on Fl was *Physarum*, including 11 species, accounting for 26.83% of the total number of species on Fl. There were also nine species in the genus *Didymium*, and the percentage (21.95%) was relatively more significant. There were seven species of the dominant genus *Didymium* fruiting on Others, accounting for 21.88%. Species were most abundant on Dw, followed by Fl, Others, Ba, and Br. The Shannon-wiener index in descending order was Dw, Others, Fl, Br, and Ba. As the species rank curve shows (Fig 4, S2 Table), the species richness of myxomycetes was highest on Dw, but there were dominant species in each substrate type.

### Distribution of myxomycete species on different substrates

**Fig 5:**
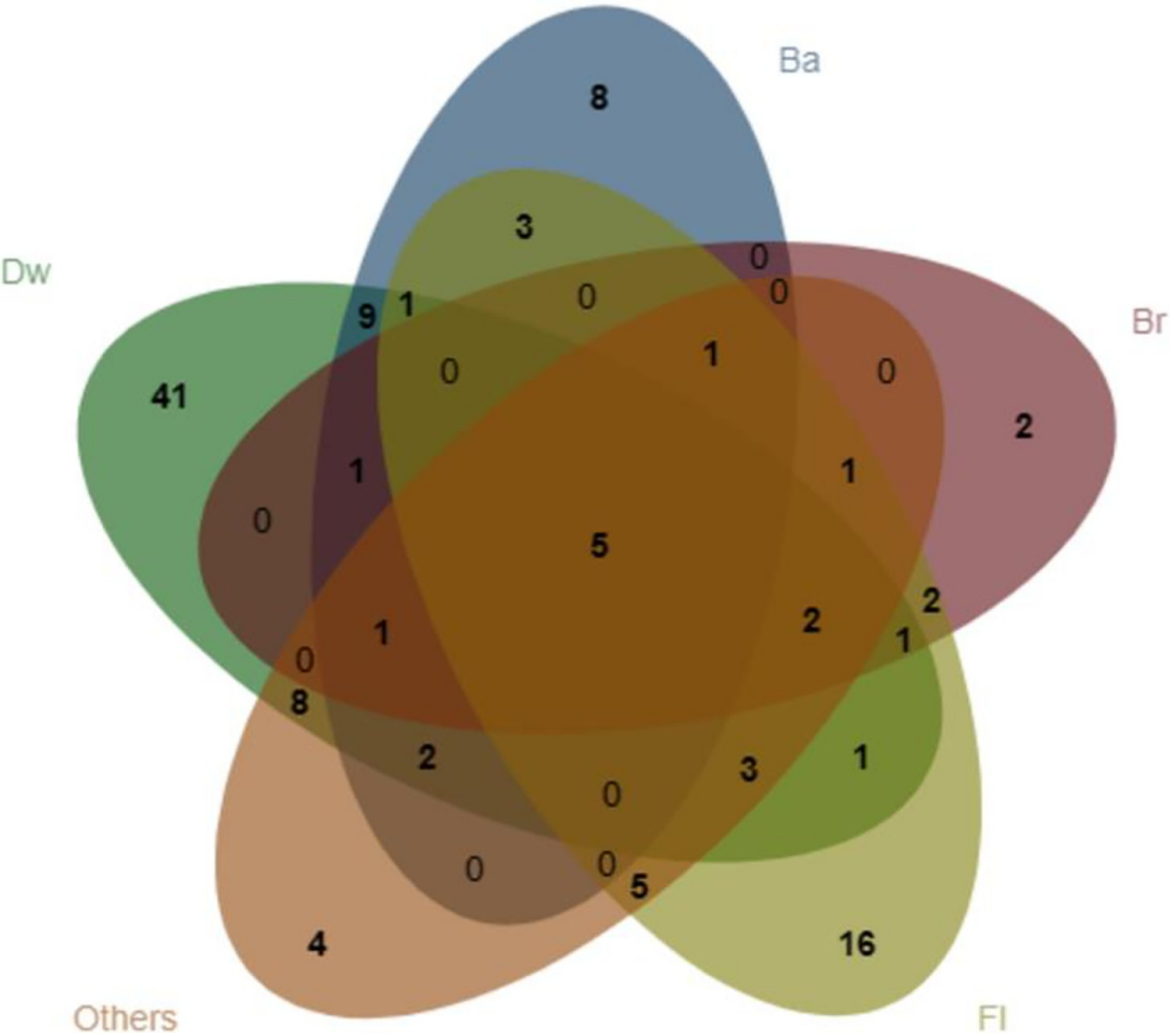
Venn diagram of myxomycetes among different substrate types.

The unique species found on different substrates are detailed in Fig 5 (S3 Table) and Table 6. Seventy-three species were found on Dw of all the species collected; forty-one species were only found on Dw, including *Cribraria argillacea*, *Comatricha fragilis*, *Licea minima*, *Tubifera dimorphotheca*, *Badhamia macrospora*, *Fuligo septica* and *Physarum leucophaeum* et al. Thirty-one species produced fruit on Ba. *Stemonitis marjana*, *Arcyria occidentalis*, *Perichaena depressa*, *Trichia lutescens*, *Trichia scabra*, *Trichia subfusca* and *Trichia varia* were only found on Ba. With 16 species fruiting on Br, *Physarum robustum* and *Diderma rugosum* were only found on Br. With 41 species fruiting on Fl including: *Craterium minutum* var. *brunneum*, *Physarum plicatum*, *Diachea bulbillosa*, *Diderma effusum*, *Didymium flexuosum*, *Arcyria globosa* and *Perichaena vermicularis* et al., sixteen species were found on Fl only. While 32 species were observed fruiting on Others and *Comtrichia pellucida*, *Didymium comatum*, *Physarum didermoides* and *Stemonitis smithii* were observed on Others only. The unique species was most prevalent on Dw.

**Table 6:** Details of unique species on different substrate types.

Substrates	Unique species	Number
**Dw**	*C*. *argillacea*, *C*. *atrofusca*, *C*. *media*, *C*. *microcarpa*, *C*. *persoonii*, *C*. *purpurea*, *C*. *stellifera*, *C*. *tenella*, *Lic*. *minima*, *L*. *epidendrum*, *L*. *conicum*, *Tub*. *dimorphotheca*, *B*. *macrospora*, *F*. *septica*, *P*. *leucophaeum*, *P*. *roseum*, *W*. *reticulata*, *Did*. *radiatum*, *D*. *crustaceum*, *Com*. *fragilis*, *Com*. *laxa*, *Com*. *nigra*, *E*. *papillatum*, *Col*. *arcyrionema*, *M*. *synsporos*, *Stem*. *fuscoides*, *Stem*. *irregularis*, *Stem*. *laxiretis*, *Stem*. *reticulospora*, *S*. *axifera*, *S*. *flavogenita*, *Ste*. *hyperopta* var. *landewaldii*, *Ste*. *typhina*, *Sym*. *flaccidus*, *A*. *denudata*, *A*. *insignis*, *A*. *obvelata*, *A*. *pomiformis*, *A*. *versicolor*, *T*. *affinis*, *T*. *decipiens*	41
**Ba**	*P*. *galbeum*, *S*. *marjana*, *A*. *occidentalis*, *Per*. *depressa*, *T*. *lutescens*, *T*. *scabra*, *T*. *subfusca*, *T*. *varia*	8
**Br**	*P*. *robustum*, *Did*. *rugosum*	2
**Fl**	*Cra*. *minutum* var. *brunneum*, *P*. *auriscalpium*, *P*. *bogoriense*, *P*. *leucopus*, *P*. *newtotii*, *P*. *oblatum*, *P*. *plicatum*, *Dia*. *bulbillosa*, *Dia*. *splendens*, *Dia*. *subsessilis*, *Did*. *effusum*, *D*. *flexuosum*, *D*. *leoninum*, *D*. *verrucosporum*, *A*. *globosa*, *Per*. *vermicularis*,	16
**Others**	*P*. *didermoides*, *D*. *comatum*, *Com*. *pellucida*, *S*. *smithii*	4

### Common species on the different substrates

We investigated common species isolated from the different substrates (Table 7). Of all species: *Cribraria aurantiaca*, *Comatrichia tenerrima*, *Stemonaria longa*, *Stemonitis fusca*, *Stemonitis splendens*, *Arcyodes incarnata*, *Arcyria incarnata*, *Trichia favoginea* and *Trichia persimilis*, nine species fruited on Dw and Ba, *Trichia botrytis* on Dw and Fl, *Cribraria cancellata*, *Cribraria mirabilis*, *Cribraria piriformis*, *Cribraria vulgaris*, *Tubifera ferruginosa*, *Tubifera montana*, *Lepidoderma tigrinum* and *Hemitrichia clavata* on Dw and Others (Mosses or Litters), *Craterium aureum*, *Physarum melleum* and *Arcyria oerstedi* on Dw, Fl, and Others (Mosses or Litters), *A*. *cinerea* on Dw, Ba, and Br, *Lamproderma scintillans* on Dw, Ba, and Fl, *Physarum album* on Dw, Ba, Br, and Others (Mosses), *Cribraria splendens* and *Hemitrichia calyculata* on Dw, Ba, and Others (Mosses), *Craterium minutum* and *Didymium squamulosum* on Dw, Br, Fl, and Others (Stones and Pine needles). Additionally, *Craterium leucocephalum* var. *cylindricum*, *Physarum ovisporum*, *Didymium columellacavum*, *Didymium difforme*, and *Didymium minus* were found on Fl and Others (Litters and Pine needles), *Tubifera applanata*, *Craterium reticulatum* and *Physarum citrinum* colonized Ba and Fl, *Didymium megalosporum* were observed on Ba, Br, Fl, and Others (Pine needles and Stones), *Didymium hemisphaericum* and *Com*. *pulchella* was noticed on Br and Fl, *Craterium leucocephalum* was found on Br, Fl, and Others (Pine needles, Litters, and Grasses), *Leocarpus fragilis*, *Diachea leucopodia*, *Didymium nigripes*, *P*. *viride* and *Hemitrichia serpula* could produce fruiting bodies on every substrate (Others: Grasses, Live grasses, Pine needles, Stones and Mosses). The highest common species abundance was observed on Dw and Ba; five species were found on each substrate type.

**Table 7:** Common species on different substrates.

Substrates	Common species	Number
**Dw, Ba**	*C*. *aurantiaca*, *Com*. *tenerrima*, *Stem*. *longa*, *S*. *fusca*, *S*. *splendens*, *Arc*. *incarnata*, *A*. *incarnata*, *T*. *favoginea*, *T*. *persimilis*	9
**Dw, Fl**	*T*. *botrytis*	1
**Dw, Others**	*C*. *cancellata*, *C*. *mirabilis*, *C*. *piriformis*, *C*. *vulgaris*, *Tub*. *ferruginosa*, *Tub*. *montana*, *Lep*. *tigrinum*, *H*. *clavata*	8
**Dw, Br, Fl**	*P*. *bivalve*	1
**Dw, Fl, Others**	*Cra*. *aureum*, *P*. *melleum*, *A*. *oerstedi*	3
**Dw, Ba, Br**	*A*. *cinerea*	1
**Dw, Ba, Fl**	*Lam*. *scintillans*	1
**Dw, Ba, Br, Others**	*P*. *album*	1
**Dw, Ba, Others**	*C*. *splendens*, *H*. *calyculata*	2
**Dw, Br, Fl, Others**	*Cra*. *minutum*, *D*. *squamulosum*	2
**Fl, Others**	*Cra*. *leucocephalum* var. *cylindricum*, *P*. *ovisporum*, *D*. *columellacavum*, *D*. *difforme*, *D*. *minus*	5
**Ba, Fl**	*Tub*. *applanata*, *Cra*. *reticulatum*, *P*. *citrinum*,	3
**Ba, Br, Fl, Others**	*D*. *megalosporum*	1
**Br, Fl**	*Did*. *hemisphaericum*, *Com*. *pulchella*	2
**Br, Fl, Others**	*Cra*. *leucocephalum*	1
**Common to all substrate types**	*Leo*. *fragilis*, *Dia*. *leucopodia*, *D*. *nigripes*, *P*. *viride*, *H*. *serpula*	5

### Species composition and similarity analysis of myxomycetes at different forest types

#### Genera of myxomycetes at different forest types

**Fig 6:**
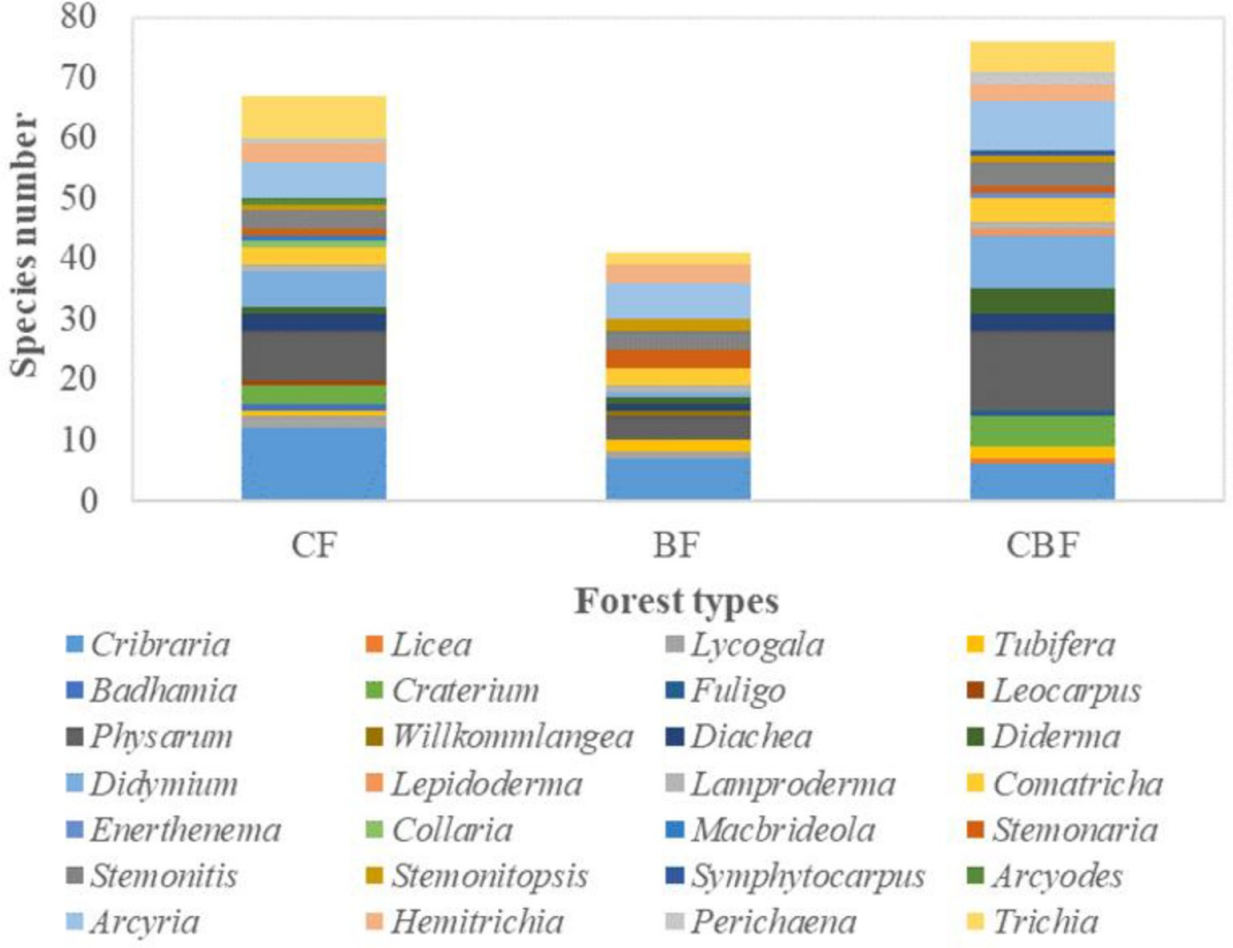
The genus level distribution at different forest types.

The species number of different genera of myxomycetes at different forest types appears in Fig 6 (S4 Table), and the dominant genus is shown in Table 8. *Cribraria* was the dominant genus in CF, including 12 species, accounting for 17.91% of species in this forest type. *Physarum* and *Trichia* had more than seven species (>10.00%) in each genus in CF. *Cribraria* also had the highest number of species in BF, with seven species accounting for 17.07%, while *Arcyria* had six species in this forest type (>10.00%). *Physarum* was the dominant genus in CBF, with 13 species accounting for 17.11%; there were also eight and nine species in *Arcyria* and *Didymium* (>10.00%) at this forest type, respectively.

**Table 8:** Statistics of dominant genera in different forest types.

Forest types	Dominant genus	Species number	Genus number	Total species	Percent (%)	S/G	*H’*	*E*
**CF**	*Cribraria*	12	22	67	17.91	3.05	3.81	0.91
**BF**	*Cribraria*	7	16	41	17.07	2.56	3.32	0.90
**CBF**	*Physarum*	13	21	76	17.11	3.62	3.84	0.89

#### The composition of myxomycetes at different forest types

**Fig 7:**
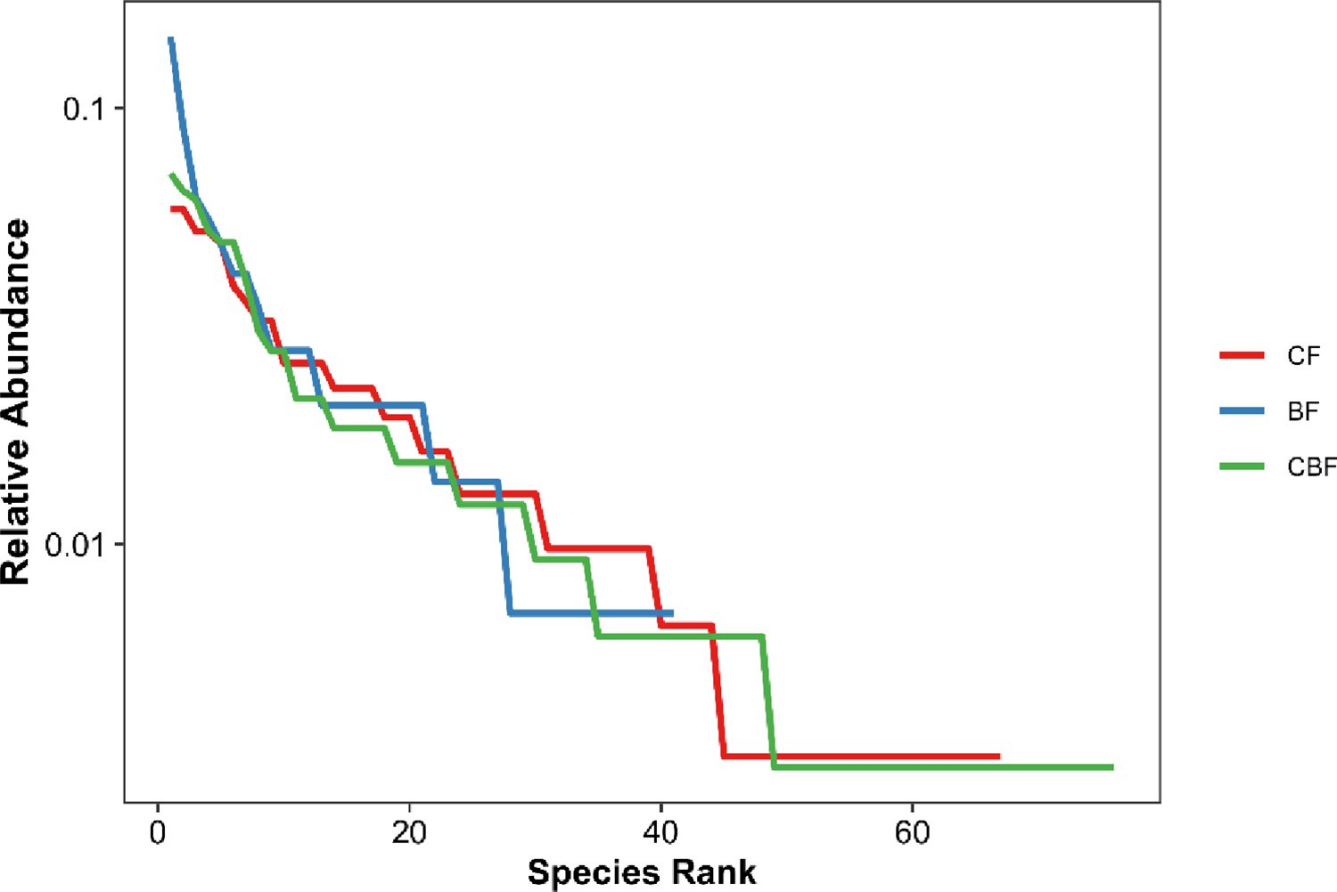
The species rank curve of different forest types.

Similarity analyses of species diversity among the collection sites with different CF, BF and CBF forest types were conducted (Table 8). Sixty-seven species were found across all collection sites in CF, belonging to 22 genera. Forty-one species of myxomycetes were found in BF, belonging to 16 genera. In the type of CBF, we found 76 species of myxomycetes belonging to 21 genera. Although the S/G (2.56) of BF was lower than CF (S/G = 3.05) and CBF (S/G = 3.62), the *H’* was also the smallest. Consequently, higher species diversity was observed in CBF (*H’* = 3.84), while abundant species might not be found in BF. In the species rank curve (Fig 7, S2 Table), the species richness of myxomycetes was the highest in the type of CBF; there were dominant species in all three forest types due to the high Pielou value (*E*).

#### Species similarity among different forest types

**Fig 8:**
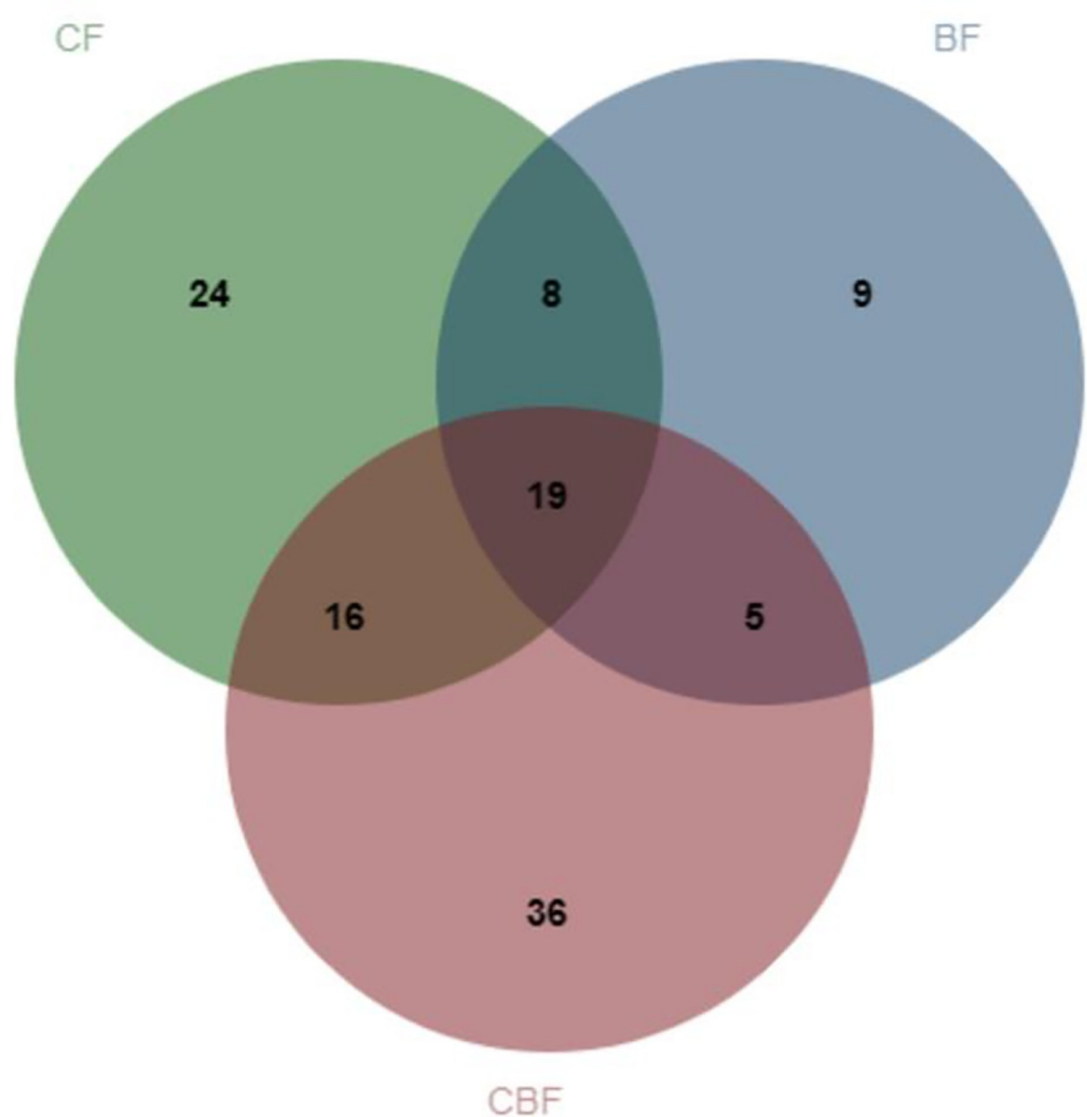
Venn diagram of myxomycetes among different forest types.

We analyzed the species similarity in different forest types, and the results are presented in Fig 8 (S5 Table), Tables 9 and 10. There were 11 common genera which consisted of 27 species in CF and BF; the similarity coefficient was 0.50. Thirty-five common species belonged to 15 genera shared between CF and CBF, with a similarity coefficient of 0.49. The similarity coefficient of species between BF and CBF was 0.41, with 12 common genera containing 24 species. The similarity coefficient between CF and BF was higher than CF and CBF, BF and CBF. There were more unique species in CBF. Nineteen species belonging to 10 genera were shared at the three forest types, viz. *Cribraria*, *Physarum*, *Didymium*, *Lamproderma*, *Comatricha*, *Stemonitis*, *Stemonitopsis*, *Arcyria*, *Hemitrichia*, *Trichia*, the distribution of species in all these genera were more extensive.

**Table 9:** Species composition and similarity in different forest types.

Forest types	Common species number	Common genus number	CC
**CF/BF**	27	11	0.50
**CF/CBF**	35	15	0.49
**BF/CBF**	24	12	0.41

**Table 10:** Common species in different forest types.

Forest types	Common species	Number
**CF/BF**	*C*. *argillacea*, *C*. *piriformis*, *C*. *vulgaris*, *Lyc*. *epidendrum*, *Coma*. *nigra*, *S*. *flavogenita*, *A*. *incarnata*, *T*. *botrytis*	8
**CF/CBF**	*Tub*. *ferruginosa*, *Cra*. *leucocephalum*, *Cra*. *minutum*, *P*. *bivalve*, *P*. *ovisporum*, *Dia*. *bulbillosa*, *Dia*. *leucopodia*, *Did*. *effusum*, *D*. *columellacavum*, *D*. *megalosporum*, *D*. *squamulosum*, *A*. *obvelata*, *A*. *versicolor*, *Per*. *depressa*, *T*. *favoginea*, *T*. *persimilis*	16
**BF/CBF**	*Dia*. *splendens*, *Did*. *hemisphaericum*, *Com*. *tenerrima*, *S*. *splendens*, *A*. *oerstedii*	5
**CF/BF/CBF**	*C*. *aurantiaca*, *C*. *cancellata*, *C*. *mirabilis*, *C*. *splendens*, *P*. *album*, *P*. *melleum*, *P*. *viride*, *D*. *nigripes*, *Lam*. *scintillans*, *Com*. *laxa*, *S*. *axifera*, *Ste*. *typhina*, *A*. *cinerea*, *A*. *denudata*, *A*. *pomiformis*, *H*. *calyculata*, *H*. *clavata*, *H*. *serpula*, *T*. *decipiens*	19

### Species similarity of myxomycetes among river valleys in Three Parallel Rivers

#### Comparison of species similarity among different river valleys

The species distribution in the West of Nujiang River was 33, forty species in the Lancang River valley, sixty-five species in the Jinsha River valley, and 45 species in the East of Jinsha River. The species similarity among different river valleys was ranked in descending order as JRV and EJR (CC = 0.53, 29 species shared), LRV and JRV (CC = 0.46, 24 species shared), WNR and LRV (CC = 0.44, 16 species shared), WNR and EJR (CC = 0.41, 16 species shared), WNR and JRV (CC = 0.37, 18 species shared), LRV and EJR (CC = 0.27, 15 species shared). From east to west, species similarity decreased between adjacent river valleys (Table 11, S6 Table).

**Table 11:** Comparison of species similarity (lower left) and common species (upper right) among different river valleys.

Location	Species Number	West of Nujiang River (WNR)	Lancang River valley (LRV)	Jinsha River Valley (JRV)	East of Jinsha River (EJR)
**West of Nujiang River (WNR)**	33		16	18	16
**Lancang River Valley (LRV)**	40	0.44		24	15
**Jinsha River Valley (JRV)**	65	0.37	0.46		29
**East of Jinsha River (EJR)**	45	0.41	0.27	0.53	

#### NMDS analysis of substrate types, forest types and river valleys

**Fig 9:**
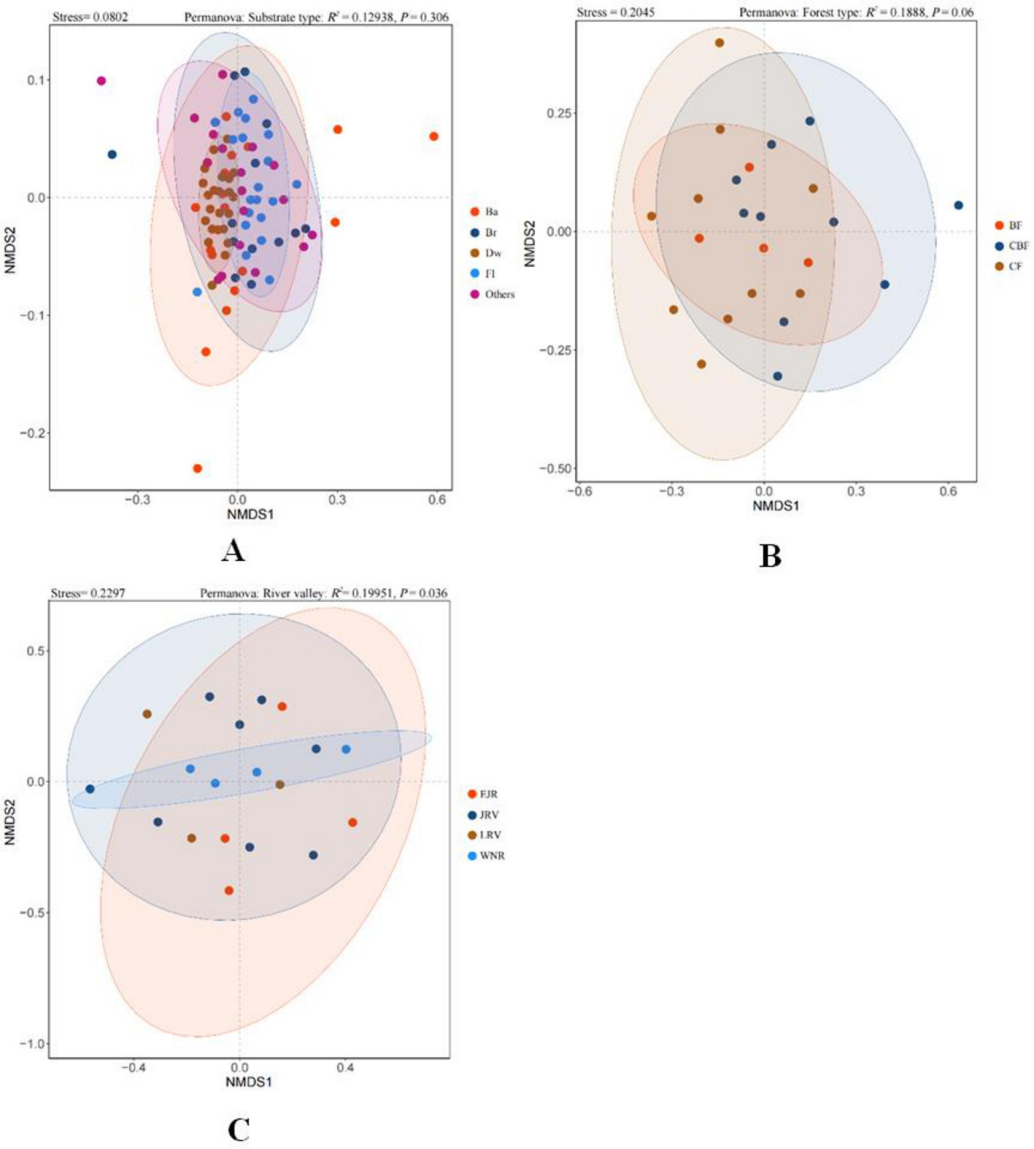
NMDS based on Bray-Curtis distances analysis of substrate type (A), forest type (B), and river valley (C). Clustering and Permanova test results are shown above.

An NMDS analysis of the myxomycete communities showed insignificant differences with substrate types (stress = 0.2045, P = 0.06; Fig 9A, S7 Table) and forest types (stress = 0.0802, P = 0.306; Fig 9B, S8 Table). In contrast, species distribution in different river valleys showed significant differences (stress = 0.2297, P = 0.036; Fig 9C, S9 Table).

## Discussion

In our result, 776 specimens of myxomycetes belonged to six orders, nine families, 28 genera, 117 species, including three varieties were identified. Four species and one variety were first recorded in China, 26 species and one variety were first recorded in Yunnan. In China, the *catalogue of Life China*: *2022 Annual Checklist* recorded 457 species [45], Song [46] added four records in 2022. In this paper, the recorded species of myxomycete in China were updated to 466. One hundred and fifty-eight records were reported in Yunnan [47–49], and the records of myxomycete in Yunnan were updated to 190. The species *Arcyria cinerea* and *P*. *viride* were the dominant species in this region. *Arcyria cinerea* was most abundant in the tropical forests of Yunnan [34, 35] and is also copious in arid deserts [2]. Some species fruit on different substrates, with a definite substrate bias; we will discuss this further, specify which species have this characteristic, and discuss the relationship between myxomycete distribution and forest type.

Species in *Diachea* almost always fruited on Fl and Br; three other species in this genus all produced fruiting bodies on Fl, but only *Dia*. *leucopodia* was found on each substrate (Table 7), which attests to the widespread distribution of this species worldwide. *Leocarpus fragilis* also was found on each substrate (Table 7), but can only be found between the 3000 and 4200 m ranges (Table 3). Also, there are no reports of this species in the tropical forests of southern Yunnan [34, 35]. This is consistent with the fact that *Leo*. *fragilis* rarely occurs in highly arid and tropical forests [50]. *Physarum viride*, *D*. *squamulosum* and *H*. *serpula* could fruit on at least four kinds of substrates (Table 7), demonstrating a more pronounced fruiting capability. Conversely, some species, such as *A*. *cinerea* and *D*. *squamulosum* et al., with fruiting ability, were not significantly affected by any factors [51]. Notably, *A*. *cinerea* only fruited on three kinds of substrate, but it was the dominant species in our study. *Perichaena depressa* is known to be a widespread species. However, we only found it fruiting on Ba (Table 3), while Stephenson [27] observed it fruiting on decaying fronds, and there were also reports which noted it fruiting on Dw and Br [32, 52], implying that its substrate selection maybe a more involved process. *Hemitrichia clavata* was collected from 10 sites amounting to 15 specimens, all observed on Dw; only one specimen was fruited on Dw and Moss. Wang [33] have also discovered this species on Dw in Yunnan, and Guo [34] further noted its scarce presence on Ba, Br, and Fl. Consequently, *H*. *clavata* may exhibit a more substantial substrate bias to Dw in this area. Although no absolute correlation exists between myxomycetes and their substrates, this frequency is not coincidental [26]. Species with high local abundance are widely distributed and may inhabit more micro-habitats, although their colonization ability depends to some extent on their ability to form fruiting bodies and micro-environmental conditions [51].

Dw seems to be the most “popular” in all the substrates in this study; similar prior findings corroborate that decaying wood is the most abundant substrate for fruiting [53–55], which might be related to Dw could supply appropriate nutrients for more species in this area. Vlasenko [56] thought that decaying woods had the advantages of physical and chemical properties and the number of microorganisms, which could help the growth of myxomycetes. Li [28] also agreed with this perspective. Generally, all the species in *Cribraria* could fruit on Dw, so it would appear that Dw was the most preferred substrate for species in this genus.

Macabago [57] discovered that the number of myxomycetes found on different substrates is not the same in the tropics in that they propagate on leaf litter (85.00%) more than bark (27.00%). This corroborates the study by Schnittler and Stephenson [8], who demonstrated that in the tropics, while in temperate forests, myxomycetes inhabiting bark (90.00%) outnumbered those found occupying leaf litter (70.00%). In temperate regions, more species were found on bark, with lower species richness in wood, ground litter, and dung [58]. In the Neotropics, many myxomycetes were observed fruiting on the decaying corollas and bracts of large living herbaceous inflorescences of the order Zingiberales [59]. In our study, we recorded the highest species abundance in individuals found occupying Dw, followed by Fl, which in turn had a greater species richness than Ba (Table 3); furthermore, all samples were collected from a plateaued, subtropical climate. Our results are similar to Schnittler and Stephenson [8], however, all the samples in the present study were collected from the field.

*Physarum viride*, *D*. *nigripes* and *H*. *serpula* were found on each substrate type and forest type (Tables 7 and 10), indicating that they are widely distributed and have strong adaptability. *Leocarpus fragilis* has been found only in coniferous forests (Table 3), although it can form fruiting bodies on each substrate (Table 7). Studies have shown that *Leo*. *fragilis* could form a large number of fruiting bodies on *Picea glauca* in Alaska [60]. This species is affected by factors other than the type of substrate. In this research, there were 14 species of *Cribraria* in total; 12 were found in CF (Table 8), which included many collection sites above 3000 m. In a previous study, Rojas and Stephenson [22] reported on the distribution of myxomycetes at high-altitude (>3000 m) oak forests in Costa Rica; they found that the most abundant species were *Cribraria piriformis*, *Cribraria mirabilis*, and *C*. *vulgaris*, three species all belong to *Cribraria*. This seems to indicate that the *Cribraria* tends to occur at high altitudes. The result was also consistent with Li [61], who thought that the species in *Cribraria* may prefer cold climates. In our results, Species or genus number of Liceales, Trichiales, and Stemonitidales showed dominance in coniferous forests (Fig 6); this is similar to Stephenson [62].

## Conclusions

Our results demonstrated a rich myxomycetes diversity in Three Parallel Rivers. The most preferred substrate of myxomycetes is Dw, followed by Fl, Ba, Others, and Br; the unique species was most abundant on Dw. The dominant genus on different substrates was different. Physarum was the dominant genus on Br and Fl, while *Cribraria*, *Trichia*, and *Didymium* were the dominant genera on Dw, Ba, and others, respectively. Species in *Cribraria* were most abundant in coniferous forest and broad-leaved forest, *Physarum* was the dominant genus in the mixed broadleaf-conifer forest, and species diversity was greatest in this forest type. Species similarity between coniferous and broad-leaved forests was higher than other pairwise comparison of forest types. NMDS analysis show that substrate and forest types had insignificant effects on myxomycetes communities, while river valley significantly affected myxomycetes communities. Substrate and forest types had no direct nor obligatory relationship to the distribution of myxomycetes, but a particular influence was noted at the species and genus levels. Based on the data in our research, the myxomycetes community similarity between river valleys is not related to the geographical proximity.

## Supporting information

S1 Table The genus level distribution at different substrate types. (XLSX)

S2 Table Species records in different forest types and substrates. (XLSX)

S3 Table Venn diagram of myxomycetes among different substrate types. (XLSX)

S4 Table The genus level distribution at different forest types. (XLSX)

S5 Table Unique and commom species in different forest types. (XLSX)

S6 Table Species in each river valley. (XLSX)

S7 Table Species records on different substrates at each collection site. (XLSX)

S8 Table Species records in different forest types. (XLSX)

S9 Table Species records in different river valleys. (XLSX)

## Abbreviations

RA: relative abundance
Dw: decayed woods
Fl: fallen leaves
Ba: barks
Br: branches
CF: coniferous forest
BF: broad-leaved forest
CBF: mixed broadleaf-conifer forest
